# Eschar [esʹ kahr, esʹ kǝr]

**DOI:** 10.3201/eid3106.240223

**Published:** 2025-06

**Authors:** Neha Srivastava, Kamran Zaman, Mahima Mittal

**Affiliations:** ICMR-Regional Medical Research Centre, Gorakhpur, India (N. Srivastava, K. Zaman); ICMR-National Institute of Traditional Medicine, Belagavi, India (K. Zaman); All India Institute of Medical Sciences, Gorakhpur (M. Mittal)

**Keywords:** rickettsia, typhus, scrub typhus, Orientia tsutsugamushi, vector-borne infections, ticks

Eschars, distinctive skin lesions from rickettsial infection induced by feeding ticks or mites, play a crucial role in diagnosing scrub typhus and spotted fever rickettsioses. Eschars occur at the sites where rickettsial pathogens are inoculated into the skin by an arthropod vector and typically emerge within a few (median 5) days after tick or mite bites, during the incubation period before symptom onset. 

Rickettsia-associated eschar is caused by rickettsial growth in endothelial cells, leading to thrombosis, ischemia, and necrosis of dermal tissue. The lesion initially resembles a small papule or pustule, then evolves into a central 0.5–3.0-cm ulcer covered by brown-black crust and encircled by a red halo (Figure), which can take several weeks to heal and can leave a small, depressed scar. Rickettsial eschars are typically painless, nonitchy, and frequently overlooked in patients with dark complexions, resulting in delayed diagnosis and treatment. PCR detection of pathogen DNA from eschar tissue or swab samples serves as a rapid diagnostic tool in early stages of eschar-associated rickettsial infection.

**Figure:**
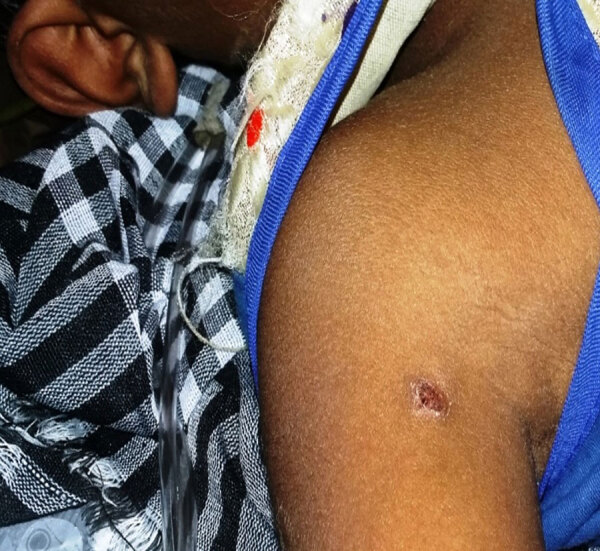
An eschar in the axillary area in the stage of healing in a patient with diagnosed scrub typhus.

Sometimes, atypical eschars resembling acne may be observed. In mite-associated rickettsioses, eschars appear in areas where skin surfaces meet or clothes bind, such as the axilla, groin, neck, and waist, but occasionally occur at uncommon sites, such as wrist or elbow joints. However, tick-associated rickettsioses often manifest eschars on the extremities and trunk. Cutaneous manifestations of other infectious diseases, including tularemia, leishmaniasis, anthrax, and certain mycobacterial and fungal infections, can also produce eschars.

The term eschar finds its root from the Ancient Greek *eskhára*, meaning hearth, brazier, or scab, from which Middle French *eschare* and Late Latin *eschara*, both signifying scar or scab, evolved. That linguistic journey reflects the lesion’s historical association with healing and scarring of burnt skin.
